# Three new species of *Cratera* Carbayo et al., 2013 from *Araucaria* forests with a key to species of the genus (Platyhelminthes, Continenticola)

**DOI:** 10.3897/zookeys.643.11093

**Published:** 2017-01-05

**Authors:** Ilana Rossi, Ana Leal-Zanchet

**Affiliations:** 1 Instituto de Pesquisas de Planárias and Programa de Pós-Graduação em Biologia, Universidade do Vale do Rio dos Sinos, 93022-750 São Leopoldo, Rio Grande do Sul, Brazil

**Keywords:** Atlantic Forest, Geoplaninae, land flatworms, Neotropical region, taxonomy, Tricladida

## Abstract

Areas of *Araucaria* moist forest have been considered to constitute hotspots of land flatworm diversity, harbouring a high number of undescribed species. Herein we describe three new species of land flatworms of *Cratera* Carbayo et al., 2013 occurring in such type of forest in south Brazil. The three species are differentiated from their congeners mainly by their colour pattern, anatomy of the pharynx and prostatic vesicle, and details of the penis papilla and male atrium. An identification key to species of the genus in the Neotropical region is provided.

## Introduction

The subfamily Geoplaninae, which has a Neotropical distribution, shows high diversity in Brazilian tropical forests (Winsor et al. 1998, Sluys 1999, Álvarez-Prezas et al. 2014). Among the phytophysiognomies which constitute the Brazilian Atlantic Forest, the mixed ombrophilous forest (*Araucaria* moist forest) has been considered to constitute hotspots of land flatworm diversity, harbouring many yet undescribed species (Leal-Zanchet and Baptista 2009, Leal-Zanchet et al. 2011). Most flatworm species described from the *Araucaria* moist forest occur in areas from its southern portion (Lemos and Leal-Zanchet 2008, Amaral et al. 2012, Leal-Zanchet et al. 2012, Lemos et al. 2014, Rossi et al. 2014, 2015).

The subfamily Geoplaninae is currently composed of 24 genera (Sluys et al. 2009, Carbayo et al. 2013), six of them recently proposed based on a combination of morphological and molecular analyses to encompass some of the species that belonged to the genus *Geoplana* Stimpson, 1857. Among them, the genus *Cratera* Carbayo, Álvarez-Presas, Olivares, Marques, Froehlich & Riutort, 2013 was proposed for five species occurring in areas of the Brazilian Atlantic Forest. Recently, another five species were described (Rossi et al. 2014, 2015; Carbayo and Almeida 2015; Negrete and Brusa 2016). Herein three new species are described, occurring in areas covered by *Araucaria* moist forest in south Brazil and a taxonomic key provided for species of *Cratera*.

## Materials and methods

Land planarians were collected in two protected areas located in the Iguassu River Drainage Basin, namely the Três Barras National Forest (26°09.27'–26°16.9'S; 50°16.0'–50°21.22'W), in Três Barras, state of Santa Catarina, and a private reserve named Araucaria Natural Heritage Private Reserve (26°20.35'–26°26.13'S; 51°19.49'–51°25.29'W), in General Carneiro, state of Paraná, both in south Brazil. They were collected during the day by direct sampling in leaf litter, under and inside fallen logs and under stones or during the night, when they are more active, by visual search.

Colour pattern and body shape and dimensions of live specimens were recorded. Specimens were then killed with boiling water and fixed in neutral formalin 10% and subsequently maintained in 70% ethyl alcohol. Methods described by Rossi et al. (2015) were used for histological processing of material and analysis of external and internal characters. The material was sectioned at intervals of 6 µm and stained with Masson’s trichrome method or haematoxylin and eosin (Romeis 1989).

Type-material is deposited in the Museu de Zoologia da Universidade do Vale do Rio dos Sinos, São Leopoldo, state of Rio Grande do Sul, Brazil (**MZU**), and the Helminthological Collection of Museu de Zoologia da Universidade de São Paulo, state of São Paulo, Brazil (**MZUSP**).

### Abbreviations used in the figures

cg
 cyanophil glands 


cmc
 common muscle coat 


cov
 common glandular ovovitelline duct 


**db** dorsal band

de
 dorsal epidermis 


df
 dorsal flecks 


di
 dorsal insertion 


dm
 dorsal cutaneous musculature 


dsm
 dorsal subcutaneous mesenchymatic musculature 


e
 eyes 


eg
 erythrophil glands 


ej
 ejaculatory duct 


es
 oesophagus 


fa
 female atrium 


fc
 female canal 


gm
 glandular margin 


go
 gonopore 


h
 halos 


i
 intestine 


im
 internal musculature 


lu
 pharyngeal lumen 


m
 mouth 


ma
 male atrium 


mas
 marginal stripe 


mes
 median stripe 


n
 nerve cord 


o
 ovary 


om
 outer musculature 


ov
 ovovitelline ducts 


p
 penis papilla 


pp
 pharyngeal pouch 


ps
 paramarginal stripe 


pv
 prostatic vesicle 


rg
 rhabditogen glands 


sg
 shell glands 


sbm
 sub-intestinal transverse mesenchymatic musculature 


sd
 sperm ducts 


sp
 sensory pit 


spm
 supra-intestinal transverse mesenchymatic musculature 


sv
 spermiducal vesicle 


t
 testes 


v
 vitelline follicles 


ve
 ventral epidermis 


vi
 ventral insertion 


vm
 ventral cutaneous musculature 


xg
 xanthophil glands 


## Taxonomy

### Family Geoplanidae Stimpson, 1857 Subfamily Geoplaninae Stimpson, 1857
*Cratera* Carbayo et al., 2013

#### Cratera cryptolineata sp. n.

Taxon classificationAnimaliaTricladidaGeoplanidae

http://zoobank.org/0A70BDC4-AA06-46E6-A8E3-3F31686E6513

##### Material examined.

Holotype: MZUSP PL.1690: *leg.* I. Rossi, 3 June 2015, Três Barras (Três Barras National Forest), state of Santa Catarina, Brazil – anterior tip: transverse sections on 12 slides; anterior region at the level of the ovaries: sagittal sections on 25 slides; pre-pharyngeal region: transverse sections on 16 slides; pharynx: sagittal sections on 30 slides; copulatory apparatus: sagittal sections on 25 slides.

Other specimens: all specimens sampled in the same locality as the holotype. MZU PL.00217: *leg.* J. A. L. Braccini, 29 July 2015 – anterior tip: transverse sections on 15 slides; anterior region at the level of the ovaries: sagittal sections on 16 slides; pre-pharyngeal region: transverse sections on eight slides; pharynx and copulatory apparatus: sagittal sections on 19 slides. MZU PL.00218: *leg.* J. A. L. Braccini, 27 July 2015 – pre-pharyngeal region: transverse sections on 14 slides; pharynx and copulatory apparatus: sagittal sections on 20 slides. MZU PL.00219: *leg.* J. A. L. Braccini, 2 June 2015 – copulatory apparatus: horizontal sections on 10 slides.

##### Diagnosis.

Species of *Cratera* with dark-brown dorsal colour, thin median stripe and greyish margins; eyes dorsal with clear halos; pharynx cylindrical; prostatic vesicle almost horizontal; penis papilla conical and symmetrical occupying distal portion of female atrium.

##### Description.

**Figure 1.**
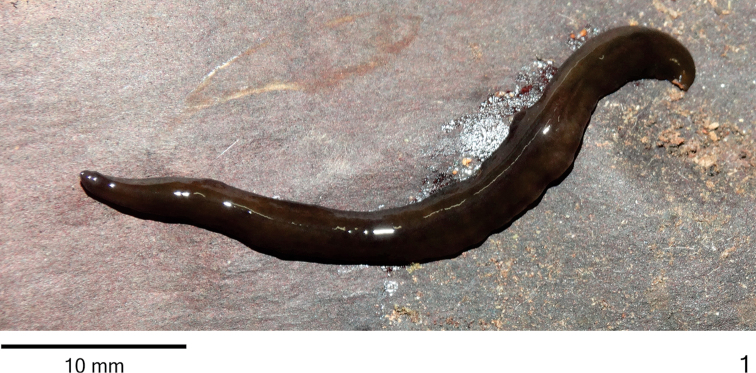
*Cratera cryptolineata* sp. n., holotype, habitus, dorsal view.

**External features.** *Body* elongate with parallel margins and dorsal surface slightly convex; anterior tip rounded and posterior tip pointed (Fig. 1). When creeping, maximum length 52mm. After fixation, maximum length 40mm (Table 1). Mouth and gonopore located at posterior fourth of body in average (Table 1).

**Table 1.** Measurements, in mm, of specimens of *Cratera cryptolineata* sp. n. Abbreviations: – not measured; * after fixation; DG distance of gonopore from anterior end; DM distance of mouth from anterior end; DMG distance between mouth and gonopore; DPVP distance between prostatic vesicle and pharyngeal pouch. The numbers given in parentheses represent the position relative to body length.

Measurement	Holotype MZUSP PL.1690	Specimen MZU PL.00217	Specimen MZU PL.00218	Specimen MZU PL.00219
Maximum length in extension	50	43	52	45
Maximum width in extension	3	2	2	3
Length at rest	25	10	30	20
Width at rest	6	5	4	7
Length*	40	35	33	34
Width*	4	3	3.5	3.5
DM*	29 (72%)	27 (77%)	25 (76%)	27 (79%)
DG*	37 (92%)	32.5 (93%)	30 (91%)	31 (91%)
DMG*	8	5.5	5	4
DPVP*	4	2.6	2.7	–
Ovaries	9 (22%)	8 (23%)	–	–
Anteriormost testes	9.5 (24%)	10 (29%)	–	–
Posteriormost testes	25.5 (64%)	23.5 (67%)	22 (67%)	–
Length of prostatic vesicle	0.4	0.3	0.3	0.35
Length of penis papilla	0.8	0.6	0.6	0.35
Length of male atrium	0.7	0.5	0.5	–
Length of female atrium	0.5	0.3	0.3	–

**Figures 2–5.**
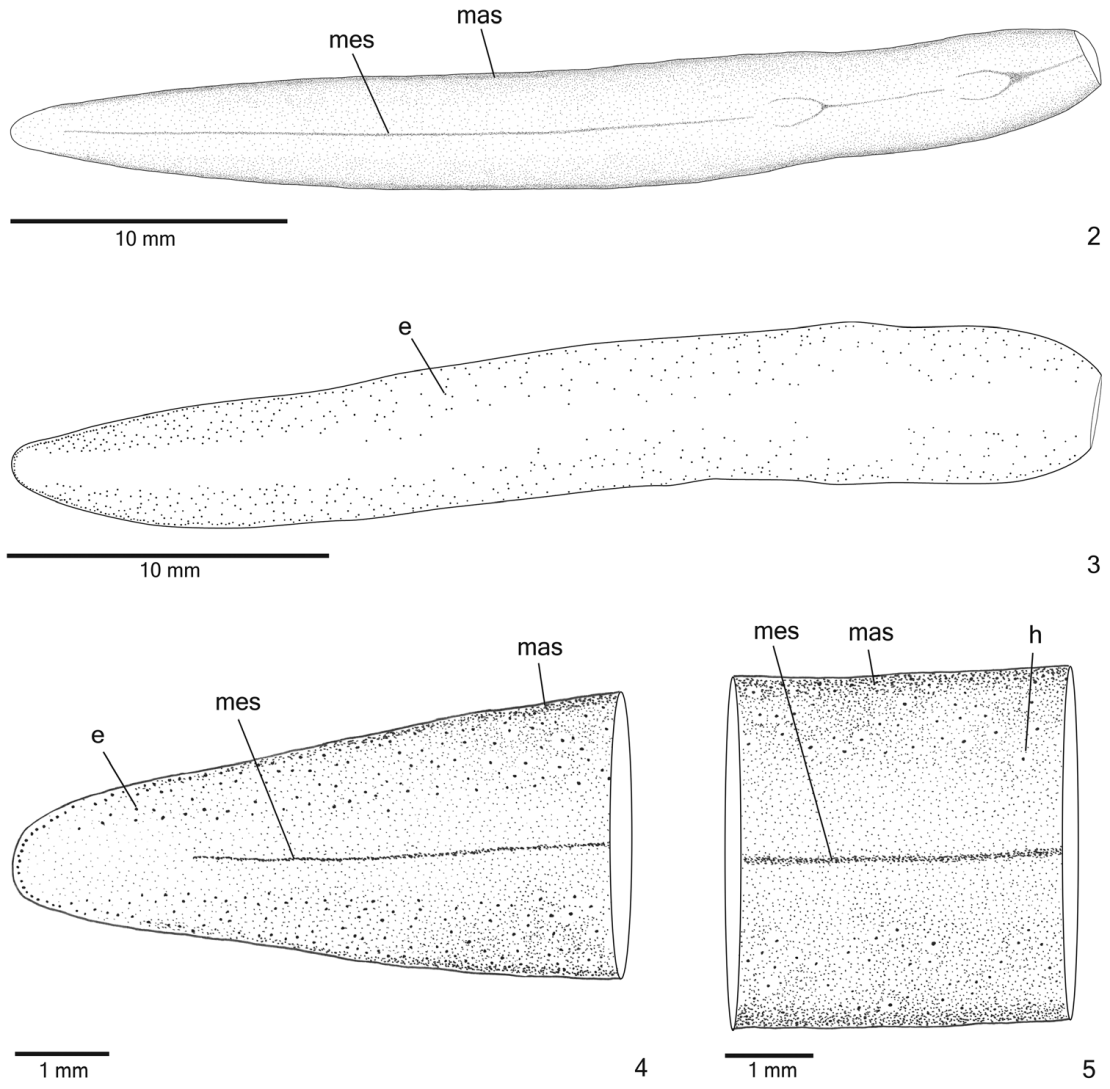
*Cratera cryptolineata* sp. n., dorsal view, **2** holotype, pattern of pigmentation **3** specimen MZU PL.00217, eye pattern **4–5** holotype, anterior extremity (**4**) and median third of body (**5**).

Live specimens with *dorsal surface* homogeneous dark-brown (Fig. 1). Under stereomicroscope, greyish ground colour visible on anterior tip, on body margins, as well as on thin median stripe occurring along body except for cephalic region. Ventral surface light brown. After fixation, dorsal pigmentation becomes light brown with darker body margins, constituting marginal stripes; thin median stripe remains greyish (Figs 2, 4–5). Ventral surface becomes light grey.

*Eyes* monolobate, initially uniserial, surround anterior tip (Figs 3–4). After first millimetre of body, eyes become larger and spread onto dorsal surface, occupying maximum width of about one-third of body width on either side of body. Eyes remain dorsal and relatively numerous towards posterior tip (Fig. 3). Inconspicuous clear halos may occur around dorsal eyes (Figs 4–5). Diameter of pigment cups 20–30 µm.

**Figures 6–13.**
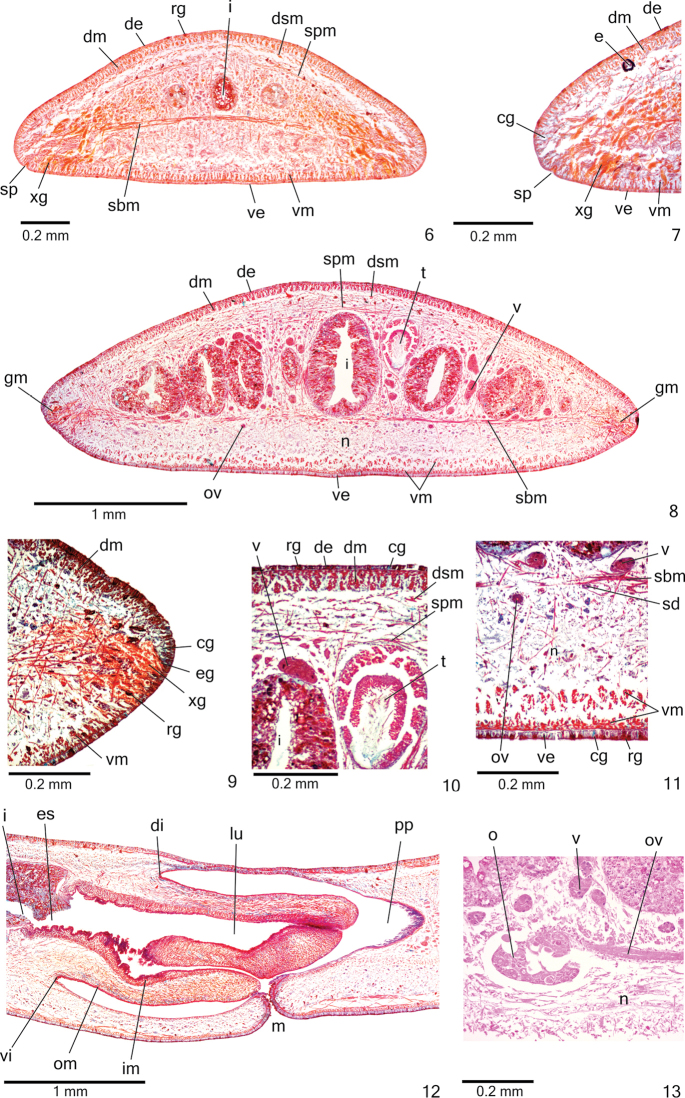
*Cratera cryptolineata* sp. n., holotype, **6–7** anterior region, transverse section **8–11** pre-pharyngeal region, transverse sections: body margin (**9**), dorsal portion (**10**), ventral portion (**11**) **12** pharynx, sagittal section **13** ovary, sagittal section.

**Sensory organs, epidermis and body musculature.** *Sensory pits* (Figs 6–7), as simple invaginations (30–40 µm deep), contour anterior tip and occur ventromarginally in irregular, single row in anterior 1/6th of body. Creeping sole occupies the whole body width in pre-pharyngeal region (Fig. 11).

Three types of glands discharge through whole epidermis of pre-pharyngeal region: rhabditogen glands with xanthophil secretion (ventrally with smaller rhabdites) and cyanophil glands with amorphous secretion, besides scarce xanthophil glands with fine granular secretion (Figs 10–11). Glandular margin (Figs 8–9) visible after first millimetre of body. At least four types of glands constitute glandular margin: numerous erythrophil glands with fine granules and xanthophil glands with coarse granules; scarcer cyanophil glands with fine granules and rhabditogen glands with xanthophil rhabdites. Glands discharging through anterior tip of body similar to those of pre-pharyngeal region (Figs 6–7).

*Cutaneous musculature* with usual three layers (circular, oblique and longitudinal layers); longitudinal layer with thick bundles (Figs 8–11, Table 2), becoming progressively lower towards body margins. Thickness of cutaneous musculature between two and five times that of epidermis (Table 2). Ventral musculature with similar thickness or slightly thicker than dorsal musculature at sagittal plane in pre-pharyngeal region (Table 2). In relation to body height, cutaneous musculature thinner in pre-pharyngeal region than in cephalic region (Table 2); thickness gradually diminishes towards anterior tip (Fig. 6).

**Table 2.** Body height and cutaneous musculature in the median region of a transversal section of the pre-pharyngeal (PP) and cephalic (CE) regions, in micrometres, and ratio of the thickness of cutaneous musculature to the height of the body (*mc:h* index) of specimens of *Cratera cryptolineata* sp. n.

Measurement	Holotype MZUSP PL.1690		Specimen MZU PL.00217		Specimen MZU PL.00218
	PP	CE	PP	CE	PP
Dorsal cutaneous musculature	50	47	43	51	45
Ventral cutaneous musculature	58	55	41	50	53
Dorsal epidermis	9	9	12	9	12
Ventral epidermis	25	12	19	12	19
Body height	1252	794	1054	818	955
*Mc:h* (%)	9	13	8	12	10

*Mesenchymal musculature* (Figs 6, 8, 10–11) well developed, mainly composed of three layers: (1) dorsal subcutaneous, located close to cutaneous musculature, with decussate fibres (3–9 fibres thick), (2) supra-intestinal transverse (5–10 fibres thick) and (3) sub-intestinal transverse (6–15 fibres thick). Mesenchymal musculature less developed in anterior region (Fig. 6) than in pre-pharyngeal region.

**Digestive system.** *Pharynx* cylindrical, nearly 5% of body length, occupies 81% of pharyngeal pouch. Pharyngeal dorsal insertion posteriorly shifted next to end of anterior third of pharyngeal pouch. Mouth slightly posterior to dorsal insertion (Fig. 12). Oesophagus short, with folded walls. Oesophagus: pharynx ratio 5%–9%.

Pharynx and pharyngeal lumen lined by ciliated, cuboidal epithelium, becoming squamous towards pharyngeal tip, with insunk nuclei. Pharyngeal glands constituted by four gland types: erythrophil glands of two types (with coarse and fine granular secretion); xanthophil glands with fine granular secretion and cyanophil glands with amorphous secretion. Outer pharyngeal musculature (4–8 µm thick) comprised of subepithelial layer of longitudinal fibres followed by layer of circular fibres. Inner pharyngeal musculature (30–40 µm thick) composed of thick subepithelial layer with circular fibres, followed by thin layer of longitudinal fibres. Both muscle layers become thinner towards pharyngeal tip. Oesophagus lined by ciliated, cuboidal to columnar epithelium with some insunk nuclei; Musculature of oesophagus (60–100 µm thick) composed of thick subepithelial layer with circular fibres, followed by thin layer with longitudinal fibres.

**Figures 14–15.**
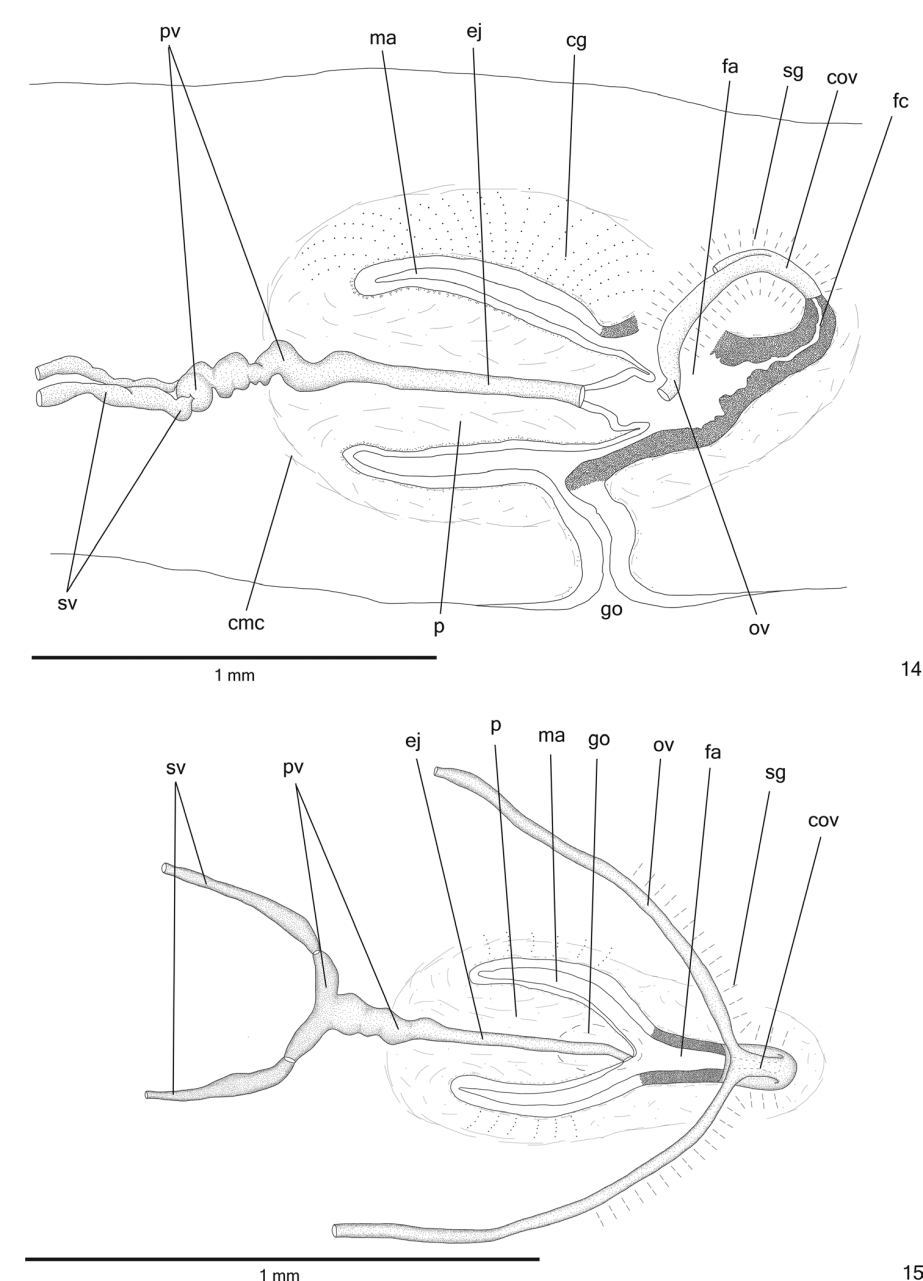
*Cratera cryptolineata* sp. n., **14** holotype, sagittal composite reconstruction of copulatory apparatus **15** specimen MZU PL.00219, horizontal composite reconstruction of copulatory apparatus.

**Figures 16–17.**
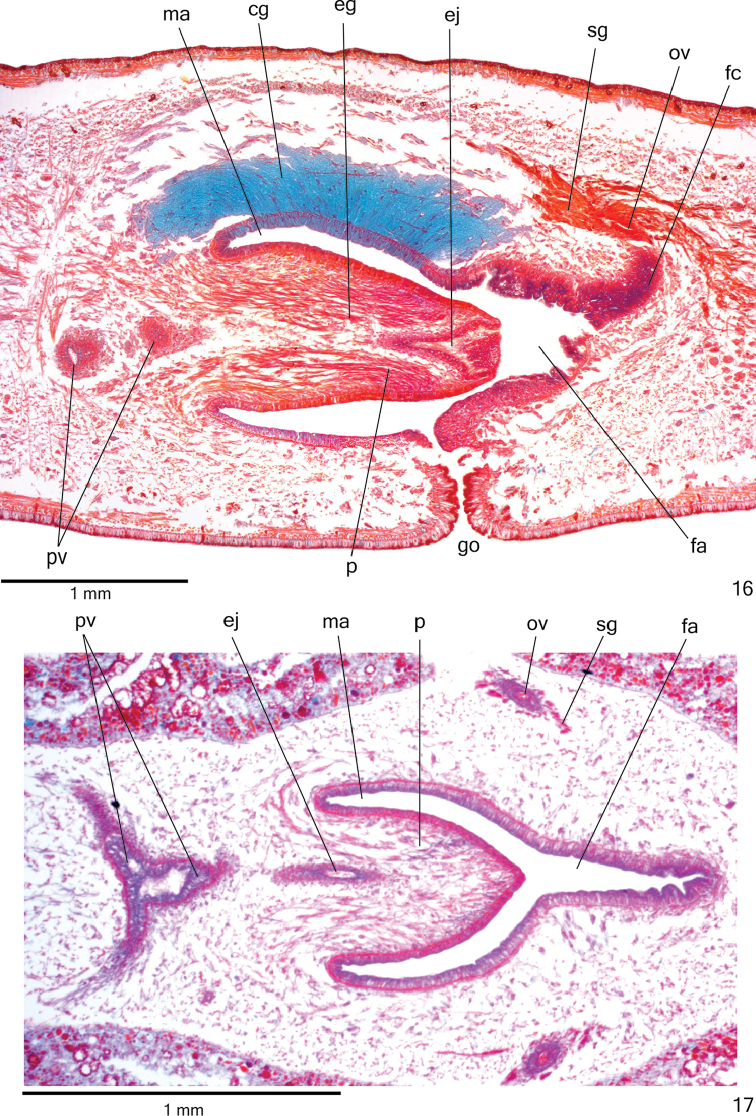
*Cratera cryptolineata* sp. n., **16** holotype, copulatory apparatus, sagittal section **17** specimen MZU PL.00219, copulatory apparatus, horizontal section.

**Figures 18–19.**
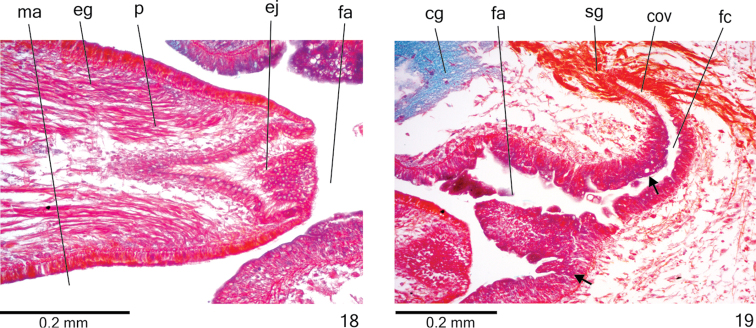
*Cratera cryptolineata* sp. n., holotype, sagittal sections, **18** penis papilla **19** female organs. Arrows indicate lacunae.

**Reproductive organs.** *Testes* in one irregular row on either side of body, located beneath dorsal transverse mesenchymal muscles, between intestinal branches (Figs 8, 10), begin slightly posteriorly to ovaries, in anterior fourth of body, and extend to near root of the pharynx (Table 1). Sperm ducts medial to ovovitelline ducts, under or among fibres of sub-intestinal transverse mesenchymal musculature, in pre-pharyngeal region (Fig. 11). They form spermiducal vesicles posteriorly to pharynx. Distally, spermiducal vesicles enter laterally into proximal portion of prostatic vesicle (Figs 14–15, 17). Extrabulbar prostatic vesicle, unpaired, located near common muscle coat, with proximal portion ample and distal portion tubular and sinuous. Proximal portion laterally expanded and T-shaped (Figs 15, 17), almost horizontal, but located closer to ventral epidermis than to dorsal epidermis (Figs 14, 16). Ejaculatory duct almost straight, opening through expansion at tip of penis papilla (Figs 14, 18). Male atrium without folds. Penis papilla conical and symmetrical, projecting into distal portion of female atrium (Figs 14–18, Table 1).

*Sperm ducts* lined with ciliated, cuboidal epithelium and coated with thin muscularis (approximately 2 µm thick) constituted of interwoven circular and longitudinal fibres. Prostatic vesicle lined with ciliated, tall columnar epithelium. Muscularis of prostatic vesicle (8–20 µm thick) comprises longitudinal and circular intermingled fibres. Ejaculatory duct lined with ciliated, columnar epithelium, with irregular height at expanded portion (Fig. 18). Muscle coat of ejaculatory duct thin (about 4 µm), mainly constituted of circular fibres. Numerous erythrophil glands with fine granular secretion as well as glands with amorphous cyanophil secretion open into both prostatic vesicle and ejaculatory duct. Penis papilla and male atrium columnar (nearly 40 µm thick) lined with non-ciliated, columnar epithelium. Xanthophil and erythrophil glands with fine granular secretion, besides glands with amorphous cyanophil secretion open through penis papilla and male atrium. Openings of cyanophil glands more numerous into male atrium and concentrated at dorso-lateral wall (Fig. 16). Muscularis of penis papilla (10–20 µm thick) and male atrium (6–10 μm thick) composed of subepithelial circular layer, followed by longitudinal layer.

*Vitelline follicles* (Figs 8, 10–11, 13) situated between intestinal branches. Ovaries ovoid (approximately 200 µm in diameter), dorsal to ventral nerve plate, in anterior fourth of body (Fig. 13, Table 1). Ovovitelline ducts emerge dorsally from median third of ovaries (Fig. 13) and run posteriorly immediately above nerve plate. Ascending portion of ovovitelline ducts located lateral to female atrium. Common glandular ovovitelline duct short, located dorsally to posterior third of female atrium (Figs 14–16, 19). Female genital duct dorso-anteriorly curved. Female atrium funnel-shaped without folds (Figs 16, 19), shorter than male atrium (Table 1).

*Ovovitelline ducts* and common ovovitelline duct lined with ciliated, columnar epithelium and covered with 5-μm-thick layer of intermingled circular and longitudinal muscle fibres. Numerous shell glands with erythrophil secretion empty into common glandular ovovitelline duct as well as into distal half of ascending portion of ovovitelline ducts (Figs 14–16, 19). Epithelial lining of female genital duct and atrium with irregular height (40–90 µm thick), stratified appearance; epithelial cells with some lacunae containing secretion (Fig. 19). Abundant cyanophil glands with amorphous secretion and erythrophil glands with fine granular secretion, as well as few xanthophil glands with fine granular secretion open into female duct and atrium. Muscularis of female duct and atrium (10–20 µm thick) composed of interwoven longitudinal and circular fibres. Specimens MZU PL.00218, MZU PL.00217 and MZU PL.00219 not fully mature, with poorly developed vitelline follicles, but showing shell glands opening into ovovitelline ducts and common glandular oviduct.

Male and female *atria* with ample communication, without separating folds (Figs 14–17). Common muscle coat thin along both male and female atria, thicker dorsally than ventrally, composed of circular, longitudinal and oblique fibres. Gonoduct vertical, lined with ciliated, columnar epithelium. Numerous cyanophil glands with amorphous secretion and rhabditogen glands with xanthophil secretion, as well as scarce erythrophil glands with fine granular secretion empty into gonoduct. Muscularis of gonoduct comprised of subepithelial layer of circular fibres, followed by longitudinal layer.

##### Etymology.

The specific name is a composite of the Greek adjective *kryptós* (hidden) and the Latin noun *linea* (stripe), referring to the thin median stripe, visible only under the stereomicroscope.

##### Distribution.

Known only from the type locality.

#### Cratera nigrimarginata sp. n.

Taxon classificationAnimaliaTricladidaGeoplanidae

http://zoobank.org/2EA2144E-2F1B-4752-9CBB-2AD34CA51633

##### Material examined.

Holotype: MZUSP PL.1691: *leg.* I. Rossi, 18 July 2015, General Carneiro (Araucaria Natural Heritage Private Reserve), state of Paraná, Brazil – anterior region in three fragments on 114 slides; pre-pharyngeal region: transverse sections on 10 slides; pharynx: sagittal sections on 19 slides; copulatory apparatus: sagittal sections on 17 slides.

Other specimens: all specimens sampled in the same locality as the holotype. MZU PL.00220: *leg.* I. Rossi, 6 February 2015 – anterior tip: transverse sections on 24 slides; anterior region at the level of the ovaries: sagittal sections on 78 slides; pre-pharyngeal region: transverse sections on 16 slides; pharynx: sagittal sections on 40 slides; copulatory apparatus: sagittal sections on 33 slides. MZU PL.00221: *leg.* J. L. A. Braccini, 4 June 2015 – copulatory apparatus: horizontal sections on 20 slides.

##### Diagnosis.

Species of *Cratera* with light-brownish dorsal colour bordered by dark margins; eyes dorsal with clear halos and bilobed appearance; pharynx cylindrical; prostatic vesicle with unbranched and dilated proximal portion; tip of penis papilla with infolds projecting into ejaculatory duct; cyanophil glands pierce male atrium evenly distributed.

##### Description.

**Figures 20–22.**
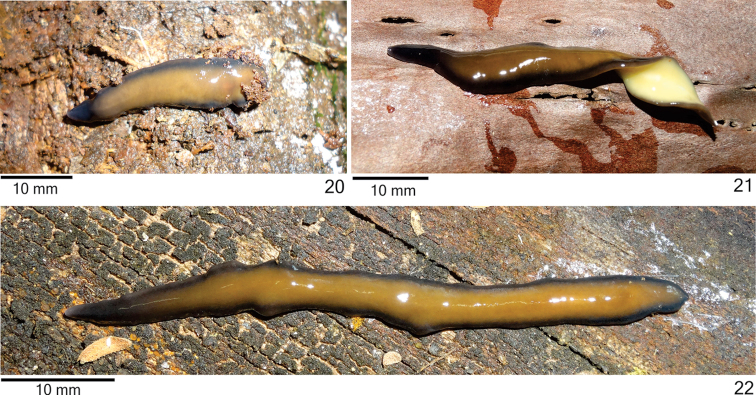
*Cratera nigrimarginata* sp. n., habitus, dorsal view, **20** holotype, anterior region **21** specimen MZU PL.00220 with part of ventral surface visible **22** specimen MZU PL.00221.

**External features.** *Body* elongate, flat and with parallel margins; anterior tip rounded and posterior tip pointed (Figs 20–22). When creeping, maximum length 57mm. After fixation, maximum length 47mm. Mouth and gonopore located at posterior fourth of body (Table 3).

**Table 3.** Measurements, in mm, of specimens of *Cratera nigrimarginata* sp. n. Abbreviations: – not measured; * after fixation; DG distance of gonopore from anterior end; DM distance of mouth from anterior end; DMG distance between mouth and gonopore; DPVP distance between prostatic vesicle and pharyngeal pouch. The numbers given in parentheses represent the position relative to body length.

Measurement	Holotype MZUSP PL.1691	Specimen MZU PL.00220	Specimen MZU PL.00221
Maximum length in extension	55	57	55
Maximum width in extension	4	4	4
Length at rest	30	46	35
Width at rest	6	5	5
Length*	46	47	45
Width*	5	5.5	4.5
DM*	35.5 (77%)	37 (79%)	37 (82%)
DG*	43.5 (94%)	42 (89%)	41 (91%)
DMG*	8	5	4
DPVP*	0.7	0.6	0.8
Ovaries	10 (22%)	11.5 (24%)	–
Anteriormost testes	13 (28%)	15.5 (33%)	–
Posteriormost testes	30.5 (66%)	33.5 (71%)	–
Length of prostatic vesicle	0.8	0.8	0.7
Length of penis papilla	1.2	1.3	1
Length of male atrium	0.9	0.9	0.9
Length of female atrium	1.2	1.6	1.2

**Figures 23–26.**
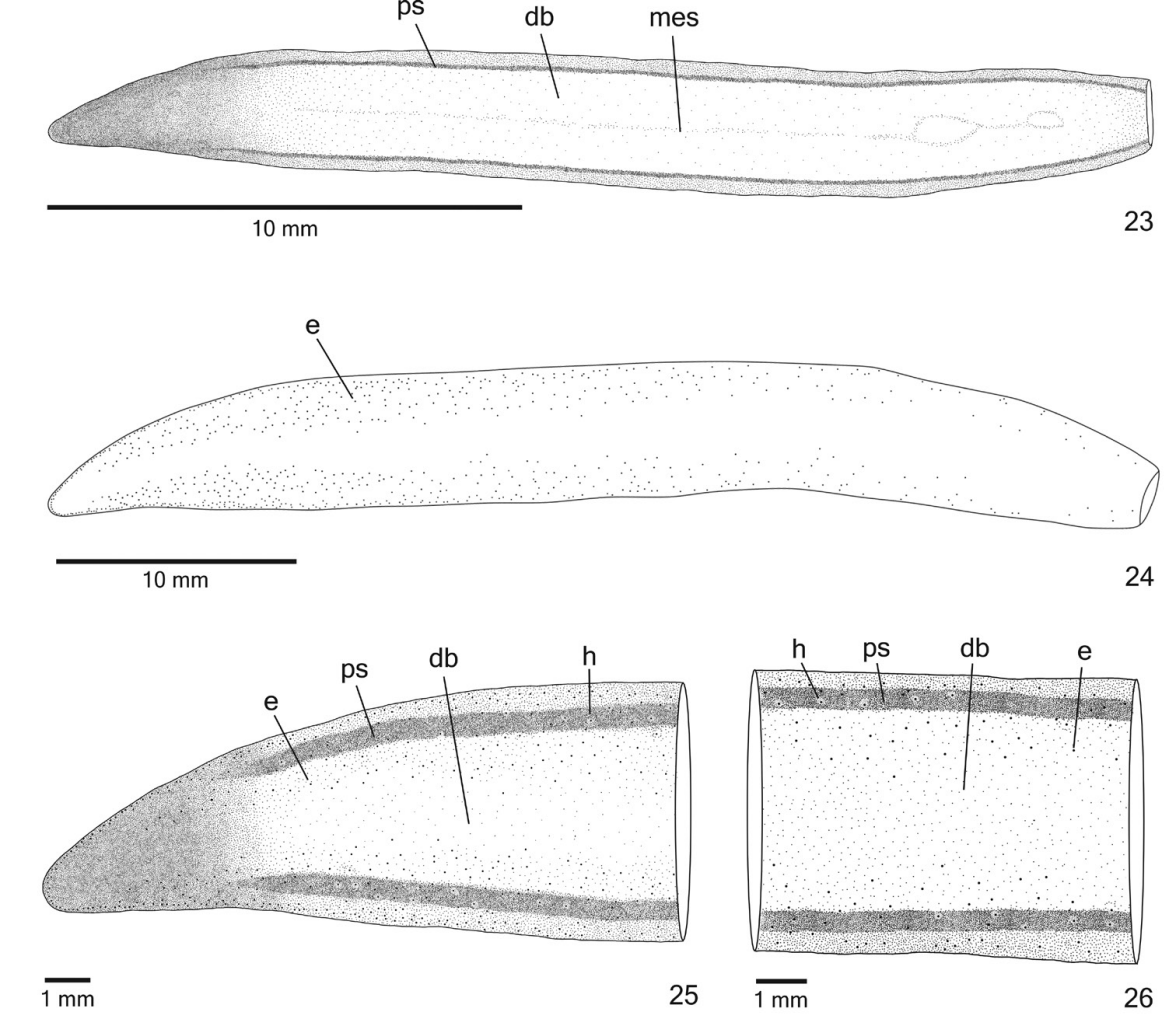
*Cratera nigrimarginata* sp. n., dorsal view, **23** holotype, pattern of pigmentation **24–26** specimen MZU PL.00220, eye pattern (**24**), anterior extremity (**25**) and median third of body (**26**).

Live animals with dorsal surface light-brownish, constituting broad band, bordered by greyish or black margins; cephalic region greyish (Figs 20–22). Ventral surface pale yellow (Fig. 21). Under stereomicroscope, dorsal band bordered by thin black paramarginal stripes. After fixation, besides dorsal band and paramarginal stripes, dorsal surface may contain inconspicuous median stripe (Fig. 23); ventral surface becomes whitish with greyish margins and anterior tip. In preserved specimens, dorsal band with maximum width of about two thirds of body width. Paramarginal stripes, with nearly 1/12^th^ of body width, begin behind the cephalic region (approximately anterior 1/9^th^ of body) and converge towards posterior tip (Figs 23, 25).

**Figures 27–34.**
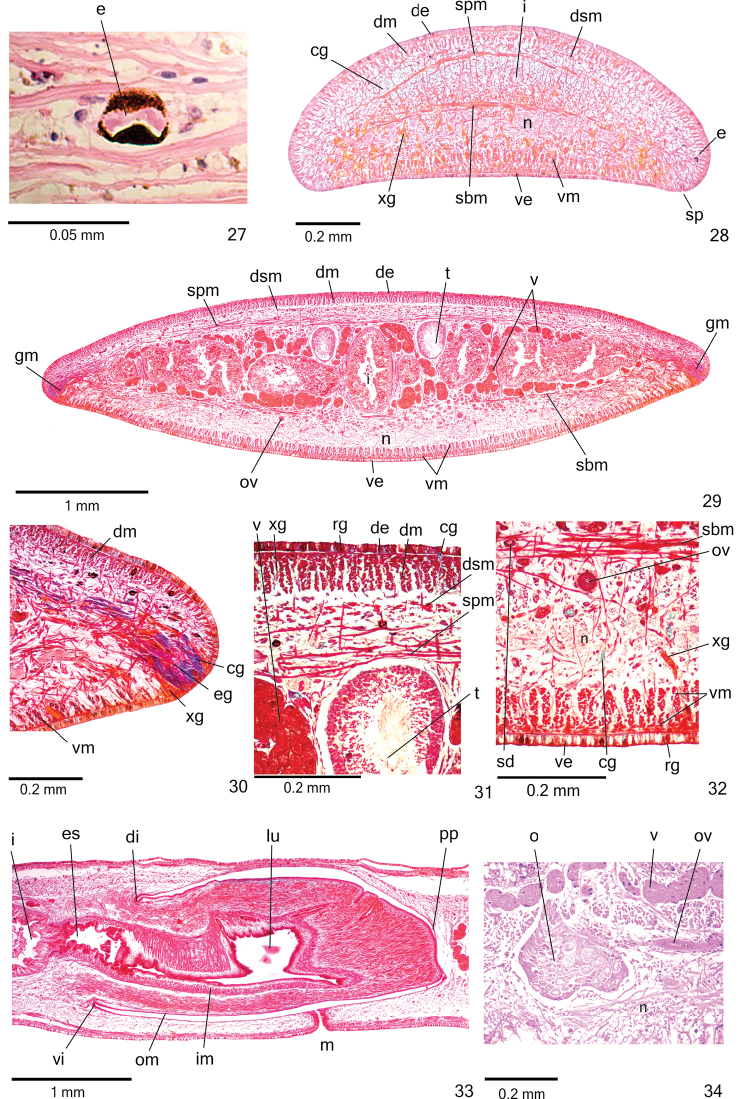
*Cratera nigrimarginata* sp. n., holotype, **27** dorsal eye, horizontal section **28** anterior region of body, transverse section **29–32** pre-pharyngeal region, transverse sections, body margin (**30**), dorsal portion (**31**), ventral portion (**32**) **33** pharynx, sagittal section **34** ovary, sagittal section.

*Eyes*, initially uniserial and monolobate, surround anterior tip (Figs 24–25). After second millimetre of body, eyes become larger and with bilobated appearance (Fig. 27), spreading onto dorsal surface and occupying almost all body width in anterior third of body (Fig. 24). Some eyes surrounded by inconspicuous small clear halos over paramarginal stripes (Figs 25–26). Eyes less numerous towards posterior tip. Diameter of pigment cups 15–40 µm.

**Sensory organs, epidermis and body musculature.** *Sensory pits* (Fig. 28), as simple invaginations (30–40 µm deep), contour anterior tip and occur ventromarginally in irregular, single row in anterior 1/8th of body. Creeping sole occupies 90% of body width in pre-pharyngeal region.

Three types of glands discharge through whole epidermis of pre-pharyngeal region: rhabditogen cells with xanthophil secretion (ventrally with smaller rhabdites), cyanophil glands with amorphous secretion and xanthophil glands with fine granular secretion (Figs 31–32). Few erythrophil glands with fine granular secretion open through ventral epidermis. Glandular margin conspicuous (Figs 29–30), after first millimetre of body. At least five types of glands constitute glandular margin: xanthophil glands with coarse granules of two types (heavily and slightly stained), cyanophil glands of two types (coarse granular and amorphous secretion) and erythrophil glands with coarse granules. Glands discharging through anterior tip of body similar to those of pre-pharyngeal region (Fig. 28).

*Cutaneous musculature* with usual three layers (circular, oblique and longitudinal layers); longitudinal layer with thick bundles (Figs 29–32, Table 4). Thickness of cutaneous musculature between two and four times that of epidermis (Table 4). Ventral musculature with similar thickness or slightly thicker than dorsal musculature at sagittal plane in pre-pharyngeal region (Table 4). Musculature becoming progressively lower towards body margins. In relation to body height, cutaneous musculature thinner in pre-pharyngeal region than in cephalic region, especially ventral musculature (Table 4, Fig. 28); thickness gradually diminishes towards anterior tip.

**Table 4.** Body height and cutaneous musculature in the median region of a transverse section of the pre-pharyngeal (PP) and cephalic (CE) regions, in micrometres, and ratio of the thickness of cutaneous musculature to the height of the body (mc:h index) of specimens of *Cratera nigrimarginata* sp. n.

Measurement	Holotype MZUSP PL.1691		Specimen MZU PL.00220	
	PP	CE	PP	CE
Dorsal cutaneous musculature	71	37	72	35
Ventral cutaneous musculature	85	85	70	77
Dorsal epidermis	15	15	19	15
Ventral epidermis	22	15	22	15
Body height	1277	484	1104	484
*Mc:h* (%)	12	25	13	23

*Mesenchymal musculature* (Figs 28–29, 31–32) well developed, mainly composed of three layers: (1) dorsal subcutaneous, located close to cutaneous musculature, with decussate fibres (6–10 fibres thick), (2) supra-intestinal transverse (8–14 fibres thick) and (3) sub-intestinal transverse (10–18 fibres thick). Mesenchymal musculature more developed in cephalic region (Fig. 28) than in pre-pharyngeal region, especially dorsal subcutaneous musculature (12–20 fibres thick).

**Digestive system.** *Pharynx* cylindrical, approximately 6% of body length, occupies 70% of pharyngeal pouch. Pharyngeal dorsal insertion slightly shifted posteriorly (Fig. 33); mouth in median third of pharyngeal pouch. Oesophagus short with folded wall. Oesophagus: pharynx ratio 4%–12%.

Pharynx and pharyngeal lumen lined by ciliated, cuboidal epithelium with insunk nuclei. Pharyngeal glands constituted by four secretory cell types: abundant erythrophil glands with fine granular secretion, xanthophil glands with coarse granular secretion, as well as two types of cyanophil glands (with amorphous and fine granular secretions). Outer pharyngeal musculature (10–30 µm thick) comprised of thin subepithelial layer of circular fibres, followed by thin layer of longitudinal fibres. Inner pharyngeal musculature (70–90 µm thick) comprises thick subepithelial layer of circular fibres, followed by thinner layer of longitudinal fibres. Outer and inner muscle layers gradually become thinner towards pharyngeal tip. Oesophagus lined by ciliated, cuboidal to columnar epithelium with insunk nuclei. Musculature of oesophagus (70–120 µm thick) composed of thick layer with circular fibres, followed by layer of longitudinal fibres.

**Figures 35–36.**
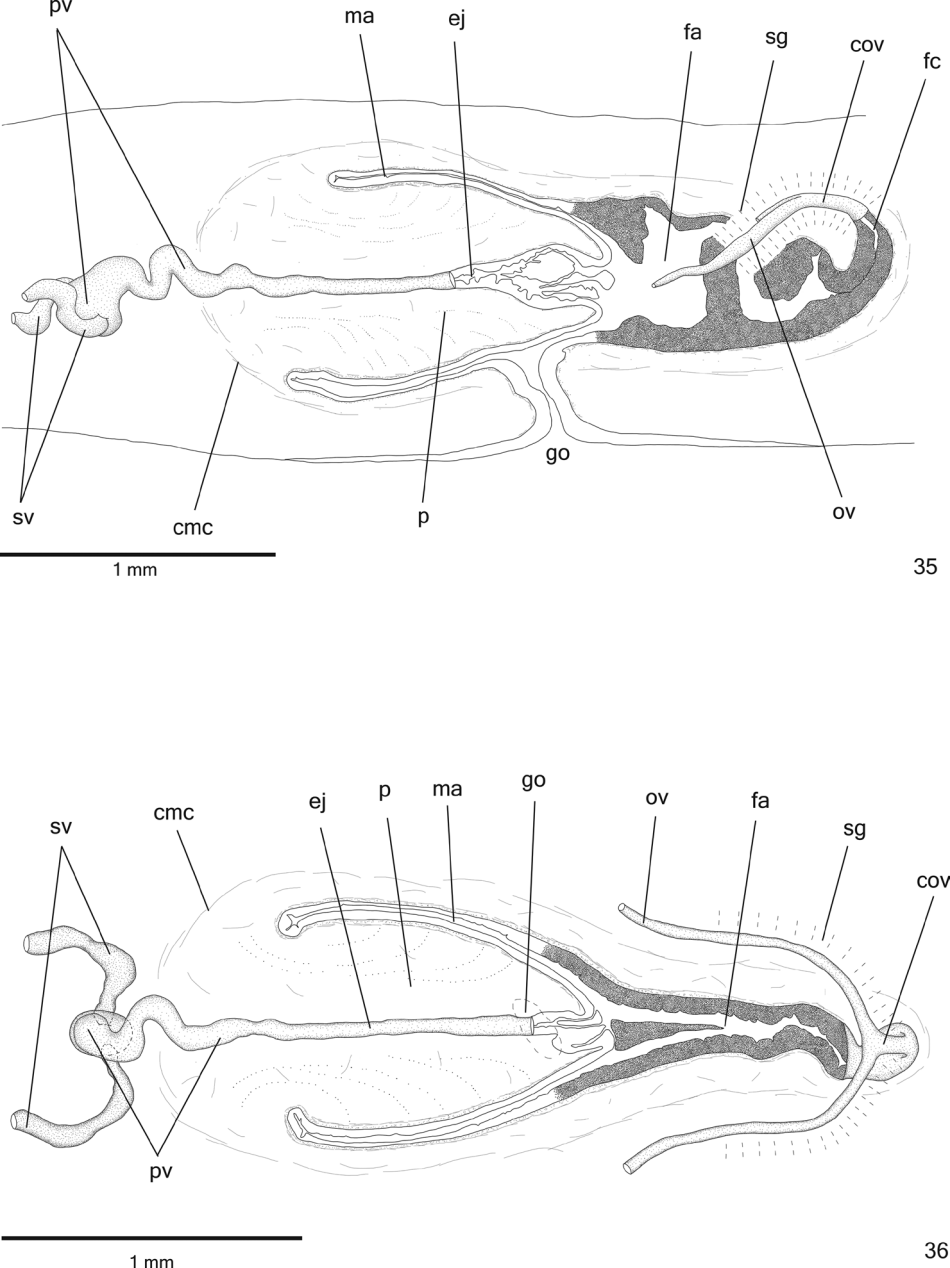
*Cratera nigrimarginata* sp. n., **35** holotype, sagittal composite reconstruction of copulatory apparatus **36** specimen MZU PL.00221, horizontal composite reconstruction of copulatory apparatus.

**Figures 37–38.**
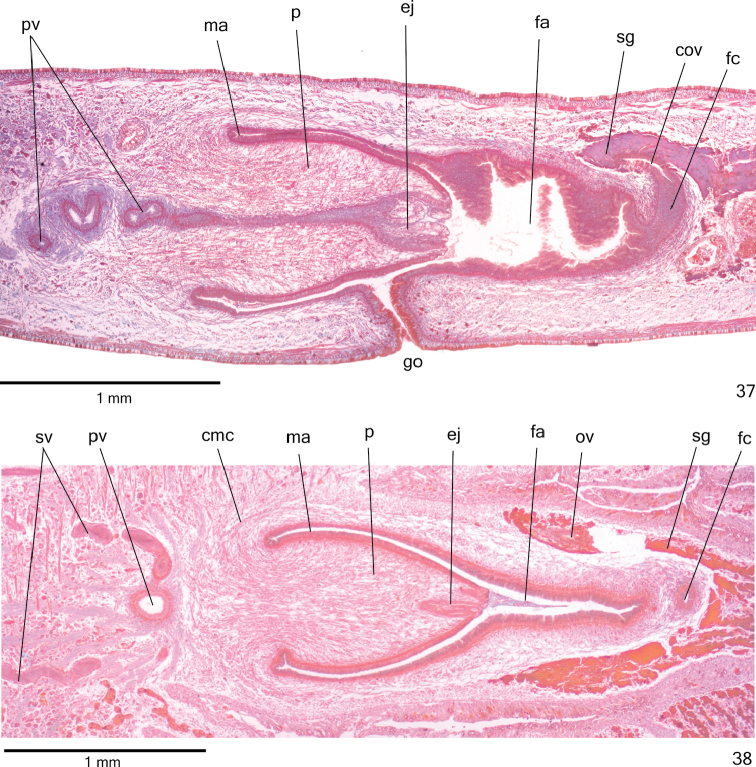
*Cratera nigrimarginata* sp. n., **37** holotype, copulatory apparatus, sagittal section **38** specimen MZU PL.00221, copulatory apparatus, horizontal section.

**Reproductive organs.** *Testes* in one irregular row on either side of body, located beneath dorsal transverse mesenchymal muscles (Figs 29, 31), begin slightly posteriorly to ovaries, in anterior third of body, and extend to near root of pharynx (Table 3). Sperm ducts medial to ovovitelline ducts, among fibres of sub-intestinal transverse mesenchymal musculature, forming spermiducal vesicles laterally to pharynx. Distally, spermiducal vesicles penetrate into lateral wall of proximal portion of prostatic vesicle (Figs 35–36, 38). Prostatic vesicle extrabulbar, unpaired, consisting of two portions: proximal portion short and dilated and distal portion tubular and sinuous. Proximal portion displaced ventrally in relation to distal portion and located closer to ventral epidermis than to dorsal epidermis (Figs 35, 37). Prostatic vesicle of specimen MZU PL.00220 showing larger lumen, filled with secretions. Ejaculatory duct almost straight, expanding at tip of penis papilla. Male atrium without folds. Penis papilla conical and symmetrical (Figs 35–38, Table 3). Tip of penis papilla occupying distal part of female atrium; with infolds projecting into ejaculatory duct (Fig. 40).

**Figures 39–41.**
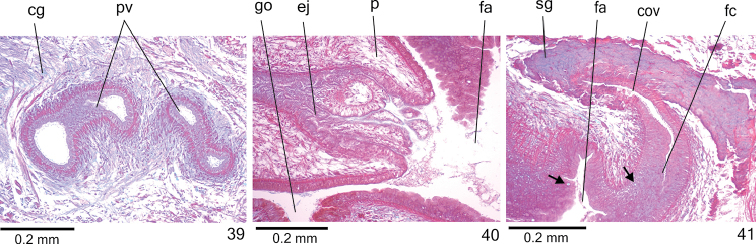
*Cratera nigrimarginata* sp. n., holotype, sagittal sections, **39** prostatic vesicle **40** penis papilla **41** female organs. Arrows indicate lacunae.

Lining epithelium of *sperm ducts* cuboidal and ciliated; thin muscularis (about 5 µm thick) constituted of interwoven circular and longitudinal fibres. Prostatic vesicle lined with ciliated, columnar epithelium. Muscularis of prostatic vesicle (20–40 µm thick) comprises mainly circular fibres mixed with longitudinal and oblique fibres (Fig. 39). Ejaculatory duct lined with ciliated, tall columnar epithelium (Fig. 40). Muscle coat of ejaculatory duct (5–10 µm) constituted of interwoven circular and longitudinal fibres. Erythrophil glands with fine granular secretion as well as cyanophil glands with amorphous secretion open into both prostatic vesicle and ejaculatory duct (Fig. 39). Penis papilla and male atrium lined with non-ciliated, columnar epithelium. Numerous cyanophil glands with amorphous secretion and few erythrophil glands with fine granular secretion open evenly distributed through penis papilla and male atrium. Muscularis of penis papilla (10–20 µm thick) and male atrium (6–10 μm thick) comprised of subepithelial layer of circular fibres, followed by layer of longitudinal fibres.

*Vitelline follicles* (Figs 29, 31, 34) situated between intestinal branches. Ovaries ovoid (approximately 200 µm in diameter) located dorsal to ventral nerve plate, in anterior fourth of body (Fig. 34, Table 3). Ovovitelline ducts emerge laterally from posterior half of ovaries and run posteriorly immediately above nerve plate. Ascending portion of ovovitelline ducts located lateral to female atrium. Common glandular ovovitelline duct short, located dorsally to posterior third of female atrium. Female genital duct dorso-anteriorly curved (Figs 35–37, 41). Female atrium oval-elongate with folded walls (Figs 35, 37), longer than male atrium (Table 3).

*Ovovitelline ducts* and common ovovitelline duct lined with ciliated, cuboidal to columnar epithelium and covered with intermingled circular and longitudinal muscle fibres (3–10 μm). Abundant shell glands with erythrophil secretion, besides cyanophil glands, empty into common glandular ovovitelline duct as well as into distal third of ascending portion of ovovitelline ducts (Figs 35–38, 41). Epithelial lining of female genital duct and atrium tall columnar, showing irregular height and sometimes stratified appearance (50–300 µm thick), ciliated in female duct. Epithelial cells with some lacunae containing cyanophil secretion (Figs 37, 41). Abundant cyanophil glands with amorphous secretion and less numerous erythrophil glands with fine granules open into female duct and atrium. Muscularis (10–20 µm thick) of female genital duct and atrium composed of interwoven circular and longitudinal fibres. Specimen MZU PL.00220 shows poorly developed vitelline follicles, but copulatory organs, including shell glands, fully developed.

Male and female *atria* broadly communicated each other, without separating folds (Figs 35–38). Common muscle coat thin along both male and female atria, thicker dorsally than ventrally, composed of circular, longitudinal and oblique fibres. Gonoduct vertical, lined with ciliated columnar epithelium. Numerous cyanophil glands with amorphous secretion and rhabditogen glands open into gonoduct. Muscularis of gonoduct comprised of thin subepithelial layer of circular fibres, followed by thin layer of longitudinal fibres.

##### Etymology.

The specific name is a composite of the Latin adjective *niger* (black) and the Latin noun *margo* (margin), referring to the colour pattern with dark margins.

##### Distribution.

Known only from its type locality.

#### Cratera aureomaculata sp. n.

Taxon classificationAnimaliaTricladidaGeoplanidae

http://zoobank.org/E4F5F32E-D05F-49C7-92A6-670A85E1D052

##### Material examined.

Holotype: MZUSP PL.1692: *leg.* J. L. A. Braccini, 3 June 2015, Três Barras (Três Barras National Forest), state of Santa Catarina, Brazil – anterior tip: transverse sections on 19 slides; anterior region at the level of the ovaries: sagittal sections on 56 slides; pre-pharyngeal region: transverse sections on 14 slides; pharynx: sagittal sections on 35 slides; copulatory apparatus: sagittal sections on 31 slides.

##### Diagnosis.

Species of *Cratera* with dorsal ground colour yellowish covered by brownish pigmentation in cephalic region and blackish pigmentation constituting irregular flecks over rest of dorsum; eyes dorsal with clear halos; pharynx cylindrical; prostatic vesicle unpaired with proximal portion displaced ventrally, laterally expanded and T-shaped; penis papilla conical and symmetrical with ventral insertion posteriorly displaced.

##### Description.

**Figure 42.**
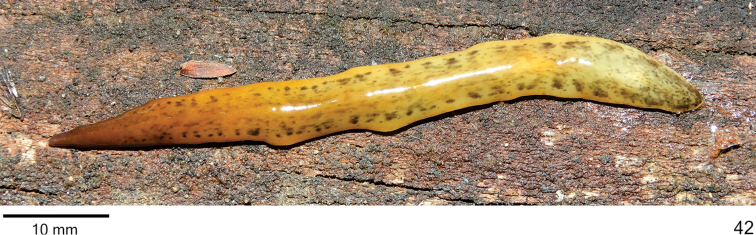
*Cratera aureomaculata* sp. n., holotype, habitus, dorsal view.

**External features.** *Body* elongate with parallel margins and dorsal surface slightly convex; anterior tip rounded and posterior tip obtuse (Fig. 42). When creeping, maximum length 55mm. After fixation, maximum length 46mm. Mouth and gonopore located at posterior fourth of body (Table 5).

**Table 5.** Measurements, in mm, of the holotype of *Cratera aureomaculata* sp. n. Abbreviations: * after fixation; DG distance of gonopore from anterior end; DM distance of mouth from anterior end; DMG distance between mouth and gonopore; DPVP distance between prostatic vesicle and pharyngeal pouch. The numbers given in parentheses represent the position relative to body length.

Measurement	Holotype MZUSP PL.1692
Maximum length in extension	55
Maximum width in extension	4
Length at rest	30
Width at rest	6
Length*	46
Width*	5
DM*	35.5 (77%)
DG*	43.5 (95%)
DMG*	8
DPVP*	4
Ovaries	12.5 (27%)
Anteriormost testes	15.5 (34%)
Posteriormost testes	30 (65%)
Length of prostatic vesicle	0.3
Length of penis papilla	0.7
Length of male atrium	0.8
Length of female atrium	0.6

**Figures 43–46.**
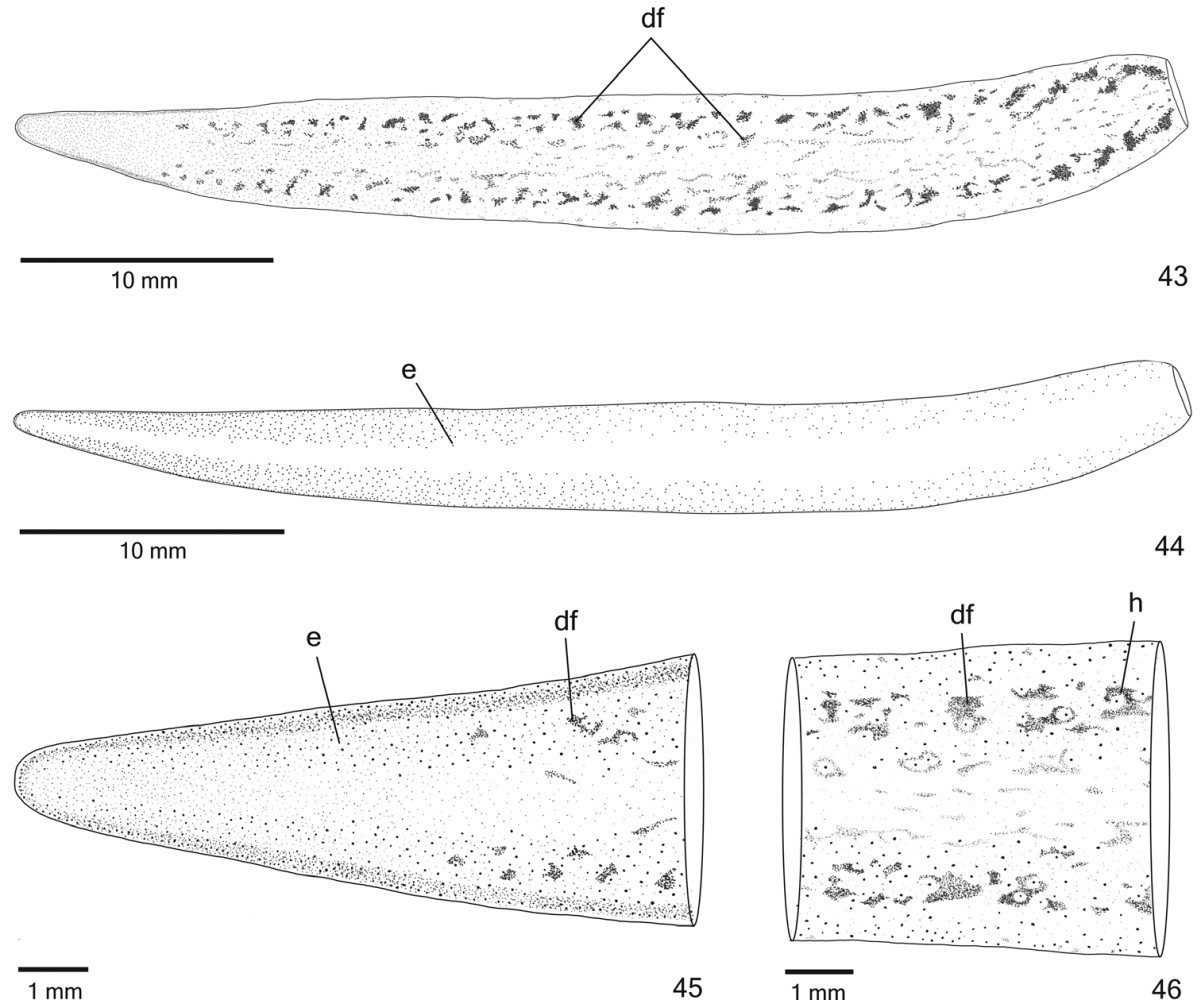
*Cratera aureomaculata* sp. n., holotype, dorsal view, **43** pattern of pigmentation **44** eye pattern **45–46** anterior extremity (**45**) and median third of body (**46**).

Live specimens with dorsal ground colour yellowish, covered by brownish pigmentation in cephalic region. Behind cephalic region, blackish pigmentation constitutes irregular flecks over dorsal surface, larger laterally and more concentrated towards posterior tip (Figs 42–43). Ventral surface light grey with yellowish margins; cephalic region (nearly anterior 1/8th of body length) brownish with darker margins.

*Eyes* monolobate, initially uniserial, surround anterior tip (Figs 44–45). After first millimetre of body, eyes become larger and spread onto dorsal surface, occupying maximum width of approximately one-third of body width on either side of body. Eyes remain dorsal, but less numerous towards posterior tip (Figs 44, 46). Some eyes over dorsal flecks surrounded by inconspicuous clear halos (Figs 45–46). Diameter of pigment cups 20–40 µm.

**Figures 47–54.**
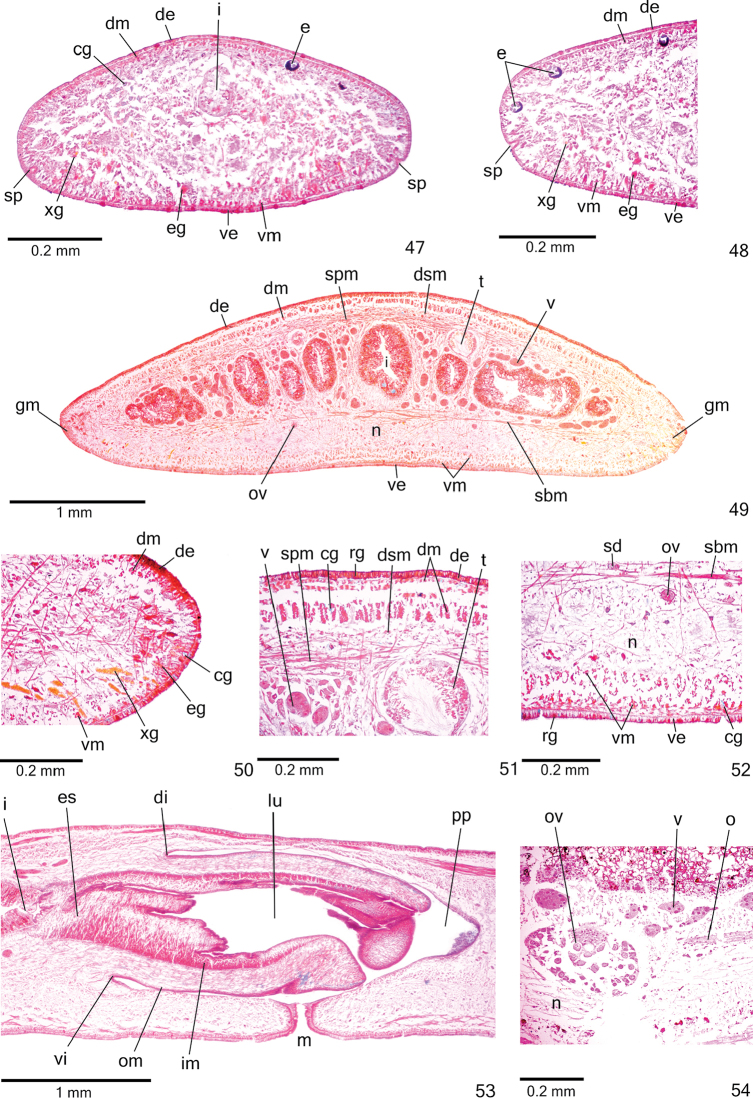
*Cratera aureomaculata* sp. n., holotype, **47–48** anterior region, transverse section **49–52** pre-pharyngeal region, transverse sections: body margin (**50**), dorsal portion (**51**), ventral portion (**52**) **53** pharynx, sagittal section **54** ovary, sagittal section.

**Sensory organs, epidermis and body musculature.** *Sensory pits* (Figs 47–48), as simple invaginations (30–60 µm deep), contour anterior tip and occur ventromarginally in irregular, single row in anterior third of body. Creeping sole occupies whole body width in pre-pharyngeal region (Fig. 52).

Three types of *glands* discharge through whole epidermis of pre-pharyngeal region: abundant rhabditogen cells with xanthophil secretion (rhammites), cyanophil glands with amorphous secretion and xanthophil glands with fine granular secretion (Figs 51–52). Glandular margin (Figs 49–50) visible after anterior 1/16th of body. At least four types of glands constitute glandular margin: xanthophil and erythrophil glands, both with coarse granular secretions, besides few xanthophil and cyanophil glands with fine granules. Glands discharging through anterior tip of body similar to those of pre-pharyngeal region (Figs 47–48).

*Cutaneous musculature* with usual three layers (circular, oblique and longitudinal layers); longitudinal layer with thick bundles (Figs 49–52, Table 6). Thickness of cutaneous musculature between four and five times that of epidermis (Table 6). Ventral musculature thicker than dorsal at sagittal plane in pre-pharyngeal region (Table 6). Musculature becoming progressively lower towards body margins. In relation to body height, cutaneous musculature slightly thinner in pre-pharyngeal region than in cephalic region (Figs 47–48), especially ventral musculature (Table 6); thickness gradually diminishes towards anterior tip.

**Table 6.** Body height and cutaneous musculature in the median region of a transversal section of the pre-pharyngeal (PP) and cephalic (CE) regions, in micrometres, and ratio of the thickness of cutaneous musculature to the height of the body (mc:h index) of the holotype of *Cratera aureomaculata* sp. n.

Measurement	Holotype MZUSP PL.1692	
	PP	CE
Dorsal cutaneous musculature	57	42
Ventral cutaneous musculature	79	50
Dorsal epidermis	15	9
Ventral epidermis	22	12
Body height	1240	719
*Mc:h* (%)	11	13

*Mesenchymal musculature* (Figs 49, 51–52) well developed, mainly composed of three layers: (1) dorsal subcutaneous, located close to cutaneous musculature, with decussate fibres variously oriented (3–5 fibres thick), (2) supra-intestinal transverse (8–14 fibres thick) and (3) sub-intestinal transverse (8–18 fibres thick). Mesenchymal musculature less developed in cephalic region (Fig. 47) than in pre-pharyngeal region.

**Digestive system.** *Pharynx* cylindrical, approximately 4% of body length, occupies 90% of pharyngeal pouch. Pharyngeal dorsal insertion slightly shifted posteriorly. Mouth in median third of pharyngeal pouch (Fig. 53). Oesophagus short with folded wall. Oesophagus: pharynx ratio 24%.

Pharynx and pharyngeal lumen lined by ciliated, cuboidal epithelium with insunk nuclei. Pharyngeal glands constituted by four secretory cell types: numerous erythrophil and xanthophil glands, both with fine granular secretion and cyanophil glands with amorphous secretion, as well as less numerous xanthophil glands with coarse granular secretion. Outer pharyngeal musculature (6–12 µm thick) comprised of thin subepithelial layer of longitudinal muscles, followed by layer of circular fibres. Inner pharyngeal musculature (60–110 µm thick) comprises thick subepithelial layer of circular fibres, followed by layer of longitudinal fibres. Outer and inner muscle layers gradually become thinner towards pharyngeal tip. Oesophagus lined by ciliated, cuboidal to columnar epithelium with insunk nuclei. Musculature of oesophagus (30–50 µm thick) composed of thick layer with circular fibres, followed by layer of longitudinal fibres.

**Figure 55.**
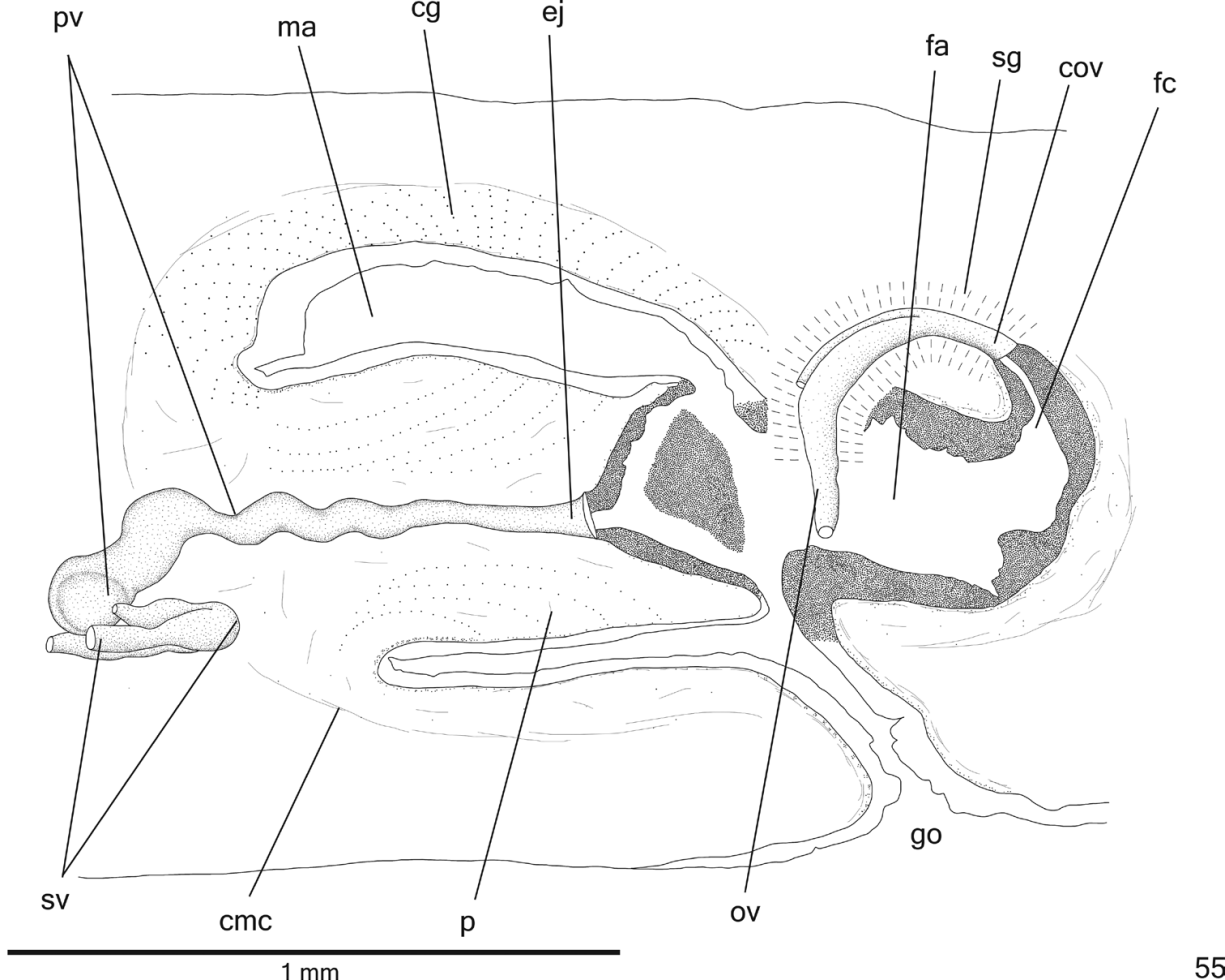
*Cratera aureomaculata* sp. n., holotype, sagittal composite reconstruction of copulatory apparatus.

**Figures 56–59.**
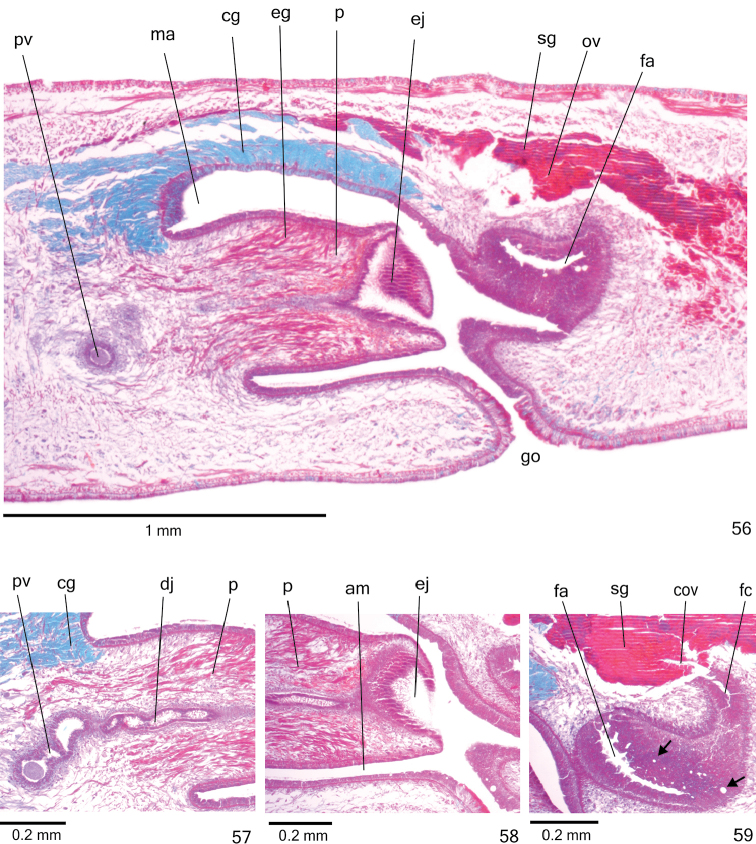
*Cratera aureomaculata* sp. n., holotype, sagittal sections, **56** copulatory apparatus **57** prostatic vesicle **58** penis papilla **59** female organs. Arrows indicate lacunae.

**Reproductive organs.** *Testes* in one irregular row in either side of body, located beneath dorsal transverse mesenchymal muscles (Figs 49, 51), begin slightly behind anterior third of body and extend to near root of pharynx (Table 5). Sperm ducts medial to ovovitelline ducts, among fibres of sub-intestinal transverse mesenchymal musculature, form spermiducal vesicles posteriorly to pharynx. Distally, spermiducal vesicles bend to enter laterally into proximal expanded portion of prostatic vesicle (Fig. 55). Prostatic vesicle extrabulbar, unpaired, located near common muscle coat, with ample proximal portion and tubular distal portion. Proximal portion laterally expanded and T-shaped, displaced ventrally in relation to distal portion and located closer to ventral epidermis than to dorsal epidermis (Figs 55–57). Ejaculatory duct with slightly sinuous proximal portion and expanded distal portion (Figs 57, 58). Male atrium without folds. Penis papilla conical and symmetrical with ventral insertion posteriorly displaced (Figs 55–56, 58, Table 5).

*Sperm ducts* lined with ciliated, cuboidal epithelium and coated with thin muscularis (about 3 µm thick) constituted of interwoven circular and longitudinal fibres. Prostatic vesicle lined with ciliated, tall columnar epithelium (Fig. 57). Muscularis of prostatic vesicle (8–20 µm thick) comprises interwoven circular, longitudinal and oblique fibres. Ejaculatory duct lined with ciliated, columnar epithelium (Fig. 58). Muscle coat of ejaculatory duct thin (about 6 µm), mainly constituted of circular fibres. Numerous glands with fine granular, mixed secretion (cyanophil external part and erythrophil internal core) empty into both prostatic vesicle and ejaculatory duct; erythrophil glands with fine granules open into ejaculatory duct. Penis papilla and male atrium lined with non-ciliated, columnar or pseudostratified epithelium (approximately 40 µm thick). Erythrophil glands with fine granules, as well as cyanophil glands with amorphous secretion open through penis papilla and male atrium, besides xanthophil glands through penis papilla (Figs 56–58); cyanophil glands concentrate their numerous openings at dorso-lateral wall of male atrium (Figs 55–57). Muscularis of penis papilla (nearly 10 µm thick) and male atrium (5–10 μm thick) composed of subepithelial circular layer, followed by longitudinal layer.

*Vitelline follicles* (Figs 49, 51, 54) situated between intestinal branches. Ovaries oval-elongate (Fig. 54), two times longer than wide (approximately 200 µm in diameter), located dorsal to ventral nerve plate, in anterior third of body (Table 5). Ovovitelline ducts emerge dorsally from median third of ovaries and run posteriorly immediately above nerve plate. Ascending portion of ovovitelline ducts located at level of gonopore. Common glandular ovovitelline duct short, located dorsally to median third of female atrium. Female genital duct dorso-anteriorly curved (Figs 55, 59). Female atrium funnel-shaped. Length of female atrium about half that of male atrium (Figs 55–56, 59, Table 5).

*Ovovitelline ducts* and common ovovitelline duct lined with ciliated, cuboidal to columnar epithelium and covered with intermingled circular and longitudinal muscle fibres (approximately 5 μm thick). Abundant shell glands with erythrophil secretion, besides cyanophil glands, empty into common glandular ovovitelline duct as well as into distal third of ascending portion of ovovitelline ducts (Figs 55–56, 59). Epithelial lining of female genital duct and atrium with irregular height and stratified appearance (30–120 µm thick); epithelial cells with some lacunae (Figs 56, 59). Abundant cyanophil glands with amorphous secretion and erythrophil glands with fine granules empty into female duct and atrium. Muscularis of female duct and atrium (8–20 μm thick) composed of interwoven circular and longitudinal fibres.

Male and female *atria* with ample communication, without separating folds (Figs 55–56). Common muscle coat thin along both male and female atria, thicker dorsally than ventrally, composed of circular, longitudinal and oblique fibres. Gonoduct anteriorly inclined, lined with ciliated columnar epithelium. Numerous cyanophil glands with amorphous secretion, besides rhabditogen glands, open into gonoduct. Muscularis of gonoduct comprised of subepithelial layer of circular fibres, followed by longitudinal layer.

##### Etymology.

The specific name is a composite of the Latin adjective *aureus* (golden) and the Latin noun *macula* (spot), referring to the colour pattern with yellowish ground colour covered by black irregular flecks.

##### Distribution.

Known only from its type locality.

### Notes on ecology and distribution

**Figures 60–61.**
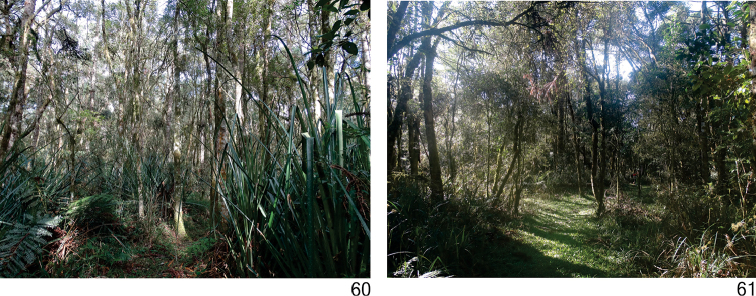
Sampling sites in type localities, **60** Três Barras National Forest, state of Santa Catarina, Brazil **61** Araucaria Natural Heritage Private Reserve, state of Paraná, Brazil.

*Cratera cryptolineata* and *Cratera aureomaculata* are sympatric in its type-locality, the Três Barras National Forest, in areas of *Araucaria* moist forest. They were recorded during night samplings in areas characterized by the dominance of *Bromelia antiachanta* Bentol. in the understorey (Fig. 60). *Cratera cryptolineata* showed high abundance in such areas, whereas *Cratera aureomaculata* was represented by a single specimen.

*Cratera nigrimarginata* occurred only in its type-locality, the Araucaria Natural Heritage Private Reserve, in a site of *Araucaria* moist forest showing an initial stage of regeneration with poorly developed understorey (Fig. 61). The species showed low abundance during both day and night samplings.

## Discussion

The three new species herein described can be easily assigned to the genus *Cratera* Carbayo et al., 2013, by presenting its diagnostic features, such as ejaculatory duct forming a distal cavity in the penis papilla, position of the ovovitelline ducts by approaching the female atrium and funnel-shaped female atrium.

By showing dorsal eyes and a cylindrical pharynx, the three species herein described share superficial similarities with four other species of *Cratera*: *Cratera joia* (Froehlich, 1956), *Cratera anamariae* Carbayo, 2015, *Cratera ochra* Rossi et al., 2015 and *Cratera viridimaculata* Negrete & Brusa, 2016 (Froehlich 1956, Carbayo and Almeida 2015, Rossi et al. 2015, Negrete and Brusa 2016), to which they are comparatively discussed.

### *Cratera cryptolineata* sp. n.

Regarding the colour pattern, by having an almost homogeneous, dark brown dorsal surface with a thin median stripe, *Cratera cryptolineata* can be differentiated from *Cratera anamariae* and *Cratera ochra*, which show a yellowish ground colour with black pigmentation forming stripes or bands (Carbayo and Almeida 2015, Rossi et al. 2015), and from *Cratera viridimaculata*, with dorsal surface stippled with dark grey fine spots on a light olive green background. The colour pattern of *Cratera cryptolineata* is similar to that of *Cratera joia*, but the latter has a broader median stripe and conspicuous clear halos surrounding eyes, whereas in *Cratera cryptolineata* clear halos are inconspicuous (Froehlich 1956).

With respect to the copulatory apparatus, *Cratera cryptolineata*, showing a penis papilla tip slightly posterior to the gonoduct, can be differentiated from *Cratera joia*, in which the penis papilla is longer, occupying half of the female atrium length. In addition, *Cratera cryptolineata* differs from *Cratera joia*, *Cratera anamariae* and *Cratera viridimaculata* by having a prostatic vesicle unforked and an almost horizontal orientation, whereas in these three species it is curved ventrally, besides being forked in *Cratera anamariae* (Froehlich 1956, Carbayo and Almeida 2015, Negrete and Brusa 2016). By showing the penis papilla with both insertions at the same transversal level, *Cratera cryptolineata* can be distinguished from *Cratera ochra* and *Cratera viridimaculata*, which show the penis papilla with the ventral insertion posteriorly displaced (Rossi et al. 2015, Negrete and Brusa 2016). The anatomy of the female atrium of *Cratera cryptolineata*, ample and without folds, also differs from that of *Cratera anamariae*, which has lateral folds (Carbayo and Almeida 2015).

### *Cratera nigrimarginata* sp. n.

By showing a light-brownish dorsal colour bordered by dark marginal stripes, *Cratera nigrimarginata* can be easily differentiated from *Cratera anamariae*, which has two broad lateral stripes, *Cratera ochra*, with dispersed pigmentation forming two broad bands, and *Cratera viridimaculata*, which show dispersed pigmentation without forming bands (Carbayo and Almeida 2015, Rossi et al. 2015, Negrete and Brusa 2016). *Cratera nigrimarginata* can also be differentiated from *Cratera joia* and *Cratera cryptolineata*, both with a light median stripe and the rest of the dorsum strongly pigmented (Froehlich 1956). In addition, *Cratera nigrimarginata* differs from their congeners by having dorsal eyes with a bilobated appearance, whereas other species show typical monolobated eyes along the body.

Regarding the copulatory apparatus, *Cratera nigrimarginata* shows an unbranched prostatic vesicle with dilated proximal portion, being differentiated from *Cratera cryptolineata*, *Cratera ochra* and *Cratera joia* with a prostatic vesicle showing proximal diverticula. In addition, it differs from *Cratera anamariae* and *Cratera viridimaculata*, which show a prostatic vesicle with forked proximal portions, C-shaped in *Cratera viridimaculata*. By having openings of cyanophil glands evenly distributed into the male atrium, *Cratera nigrimarginata* also differs from these species, in which the openings of cyanophil glands concentrate dorso-laterally into the male atrium.

### *Cratera aureomaculata* sp. n.

*Cratera aureomaculata* shows a distinctive colour pattern, showing a blackish pigmentation constituting irregular flecks over the yellowish dorsal ground colour and a brownish pigmentation in the cephalic region. Thus, it differs from stripped species, such as *Cratera nigrimarginata* and *Cratera anamariae*, as well as from species showing a strongly pigmented dorsal surface with a light median stripe, such as *Cratera joia* and *Cratera cryptolineata* (E.M. Froehlich 1955, Froehlich 1956, Carbayo and Almeida 2015). It can also be distinguished from *Cratera ochra*, which shows dispersed pigmentation forming two broad bands, and from *Cratera viridimaculata* with dark grey body margins and cephalic region (Rossi et al. 2015, Negrete and Brusa 2016).

With respect to the copulatory apparatus, *Cratera aureomaculata* shows a prostatic vesicle with proximal portion laterally expanded and T-shaped, differing from *Cratera nigrimarginata*, which has a prostatic vesicle with dilated proximal portion, as well as from *Cratera anamariae* and *Cratera viridimaculata* which show a prostatic vesicle with forked proximal portions, C-shaped in the latter. By showing the penis papilla with the ventral insertion posteriorly displaced and the proximal portion of the prostatic vesicle ventrally displaced, *Cratera aureomaculata* differs from *Cratera cryptolineata* with both insertions at the same transversal level and prostatic vesicle almost horizontal. *Cratera aureomaculata* shows the penis papilla tip anterior to the gonoduct and a common ovovitelline duct dorsal to the female atrium, being differentiated from *Cratera joia*, in which the penis papilla is longer, occupying half of the female atrium length, and a common ovovitelline duct is absent. *Cratera aureomaculata* can be distinguished from *Cratera ochra* by the position of the proximal portion of the prostatic vesicle, which is more ventrally located in relation to the rest of the vesicle in *Cratera aureomaculata* than in *Cratera ochra*, in which the prostatic vesicle is almost horizontal.

### Key to the species of the genus *Cratera* in the Neotropical region

**Table:** 

1	Colour pattern with stripes or bands	**2**
–	Colour pattern without stripes or bands	**8**
2	Eyes spreading over the dorsal surface	**3**
–	Eyes exclusively on the margins or lateral parts of the body	**9**
3	Pharynx cylindrical	**4**
–	Pharynx bell-form	**12**
4	Prostatic vesicle with proximal portion laterally expanded and T-shaped	**5**
–	Prostatic vesicle with another form	**6**
5	Dark-brown dorsal colour, with a thin median stripe and greyish margins	***Cratera cryptolineata* sp. n.**
–	Yellow-ochre dorsal colour with dispersed greyish or greyish-brown pigmentation constituting two broad dorsal bands	***Cratera ochra*Rossi et al., 2015**
6	Unbranched prostatic vesicle with dilated proximal portion	***Cratera nigrimarginata* sp. n.**
–	Prostatic vesicle with forked proximal portion or with proximal diverticula	**7**
7	Dark-greyish dorsal colour with rusty median stripe, anterior tip and margins	***Cratera joia* (Froehlich, 1956)**
–	Yellow dorsal colour with two paramedian black stripes	***Cratera anamariae* Carbayo, 2015**
8	Prostatic vesicle tubular and C-shaped with forked proximal portion	***Cratera viridimaculata* Negrete & Brusa, 2016**
–	Prostatic vesicle with proximal portion laterally expanded and T-shaped	***Cratera aureomaculata* sp. n.**
9	Short and wide penis papilla with a large intra-penial cavity	***Cratera cuarassu* Carbayo & Almeida, 2015**
–	Conical and symmetrical penis papilla without intra-penial cavity	**10**
10	Pharynx bell-form	***Cratera steffeni*Rossi et al., 2014**
–	Pharynx cylindrical	**11**
11	Orange ground colour with a light median stripe and greenish pigmentation on the anterior tip	***Cratera yara* (Froehlich, 1955)**
–	Colour pattern with four thin, black stripes, besides orange marginal bands and thin median stripe	***Cratera pseudovaginuloides* (Riester, 1938)**
12	Black dorsal surface with a thin and light median stripe	***Cratera crioula* (Froehlich, 1955)**
–	Yellowish ground colour with brownish pigmentation forming bands	***Cratera tamoia* (Froehlich, 1955)**

## Supplementary Material

XML Treatment for Cratera cryptolineata

XML Treatment for Cratera nigrimarginata

XML Treatment for Cratera aureomaculata
